# *Lithocarpus vuquangensis* (Fagaceae), a new species from Vu Quang National Park, Vietnam

**DOI:** 10.3897/phytokeys.95.21832

**Published:** 2018-01-30

**Authors:** Ngoc Nguyen Van, Hung Nguyen Viet, Binh Hoang Thi, Shuichiro Tagane, Hironori Toyama, Hoang Thanh Son, Ha Tran Viet, Tetsukazu Yahara

**Affiliations:** 1 Laboratory of Ecological Sciences, Graduate School of Systems Life Sciences, Kyushu University, 744 Motooka, Fukuoka, 819-0395, Japan; 2 Department of Biology, Dalat University, 01 – Phu Dong Thien Vuong, Dalat, Vietnam; 3 Vu Quang National Park, Ha Tinh, Vietnam; 4 Centre for Asian Conservation Ecology, Kyushu University, 744 Motooka, Fukuoka, 819-0395, Japan; 5 Silviculture Research Institute, Vietnamese Academy of Forest Sciences, Ha Noi, 10999, Vietnam; 6 Vietnam National University of Forestry, Xuan Mai, Chuong My, Ha Noi, Vietnam

**Keywords:** Fagaceae, *Lithocarpus*, new species, phylogeny, taxonomy, Vietnam, Vu Quang National Park

## Abstract

*Lithocarpus vuquangensis* Ngoc & Hung is described from Vu Quang National Park, North Central Vietnam. The morphological comparison and phylogenetic analysis based on *rbcL*, *matK* and ITS provided evidence that the new species was not assignable to any of the previously known taxa in Vietnam and its surrounding countries. The description, photographs, preliminary conservation status and DNA barcode sequences are also provided for the new species.

## Introduction

**Figure 1.**
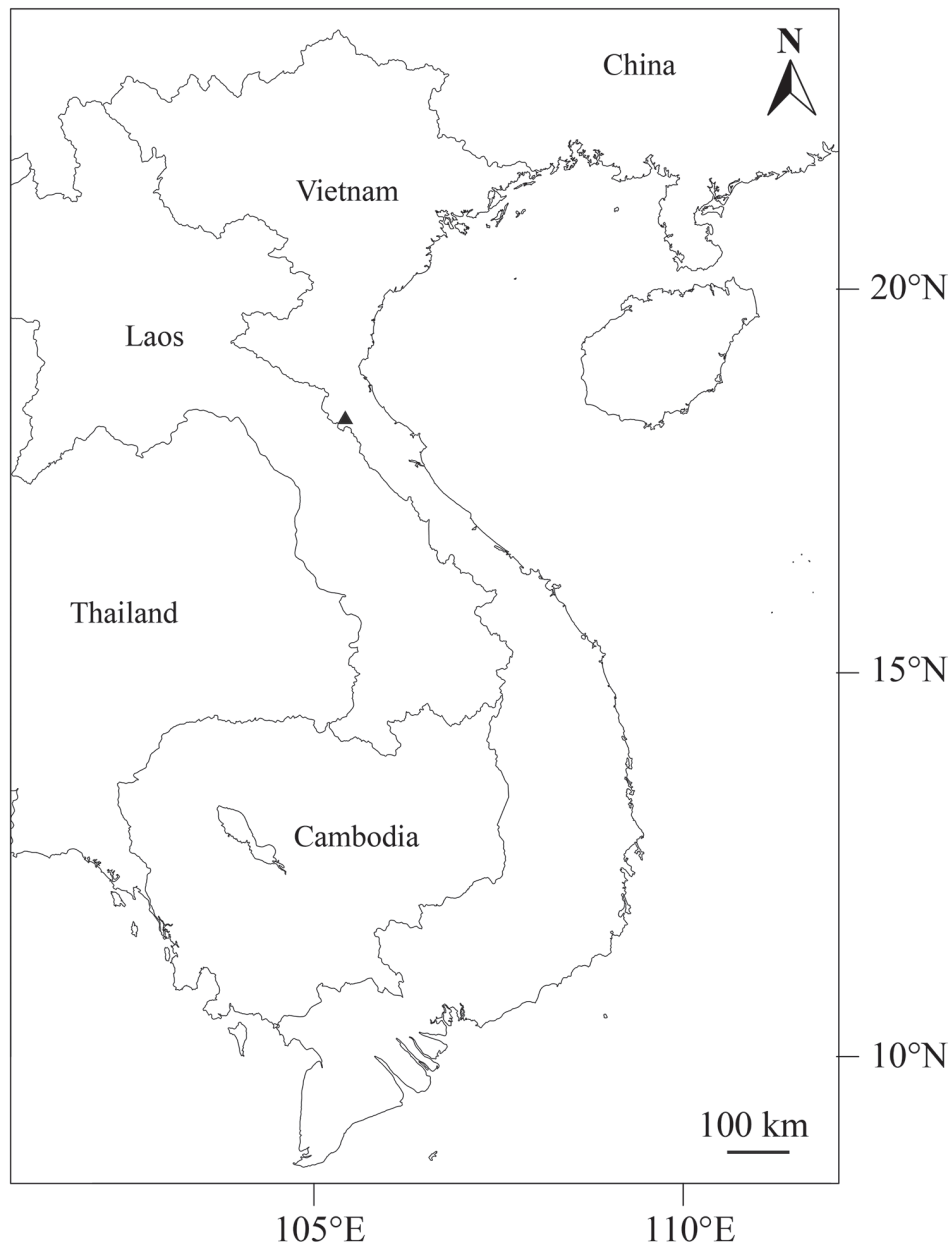
Location of Vu Quang National Park (Black triangle), type locality of *Lithocarpus vuquangensis*.

It has been known that species richness of the genus *Lithocarpus* Blume (Fagaceae Dumorier) is high in Vietnam where 120 species and two varieties have been reported including the recently published species, *L. dahuoaiensi*s Ngoc & L. V. Dung (Ban 2003, Ho 2003, Ngoc et al. 2016). Here, an additional new species of *Lithocarpus* is described from Vu Quang National Park located in Ha Tinh Province, North Central Vietnam (Figure 1).

Vu Quang National Park covers an area of ca. 56,000 ha from lowlands (alt. 10–300 m) to the highlands (the highest peak of Rao Co, alt. 2,286 m). Two new species of mammals (Sao La - *Pseudoryx nghetinhensis*, Artiodactyla and the world’s largest muntjac - *Muntiacus vuquangensis*, Cetartiodactyla) were discovered from this national park in the 1990s (Dung et al. 1993, 1994). The vegetation is diverse along the elevation gradient and five major forest types are recognised: lowland forests (alt. 10–300 m), hill forest (alt. 300–1,000 m), medium montane forest (alt. 1,000–1,400 m), montane forest (alt. 1,400–1,900 m) and upper montane forest (alt. 1,900–2,100 m) (Kuznetsov 2001, Vu Quang National Park Management Board 2014). Until now, 1,678 species of vascular plants including many endemic and rare species have been reported (Vu Quang National Park Management Board 2014, Tagane et al. 2016). As for Fagaceae, one species of *Castanea* Mill, nine species of *Castanopsis* (D. Don) Spach., 12 species of *Quercus* L. and 37 species of *Lithocarpus* Blume have been recorded from the National Park, amongst which 10 species have been listed in Viet Nam Red Data Book (Ban et al. 2007, Hung et al. 2014). In addition, natural populations of *Trigonobalanus verticillata* Forman were discovered during the authors’ recent botanical surveys in the National Park in 2016 (voucher specimens: *Yahara et al. V5764* & *V5766*, DLU, FU, the herbarium of Vu Quang National Park), which brings the number of Fagaceae genera in the region up to five.

From 2015 to 2016, floristic expeditions were carried out in Vu Quang National Park and trees of the genus *Lithocarpus* were discovered that did not match any described species. Here, the authors describe and name it as *Lithocarpus vuquangensis* Ngoc & Hung, sp. nov. accompanied with its photographs and the morphological comparison with related species. In addition to the morphological examination, DNA sequences and phylogenetic analysis are extremely helpful for identifying and delimiting species (Hebert and Gregory 2005, Dick and Webb 2012). Here, parts of the DNA barcode regions *rbcL, matK* (CBOL Plant Working Group 2009) and ITS (China Plant BOL Working Group 2011) were sequenced and the phylogenic relationship of *L. vuquangensis* and its related taxa were examined.

## Materials and methods

### Morphological observations

The morphological traits of the new species were compared with its putative relatives based on systematic literature (Camus 1948, Huang et al. 1999, Ban 2003, Ho 2003, Phengklai 2008) and more than three hundreds dried specimens kept in the following herbaria were also examined: BKF, DLU, FOF, HN, KYO, P, RUPP, TI and VNM as well as digitised plant specimen images available on the web of JSTOR Global Plants (https://plants.jstor.org/), Muséum National d’Histoire Naturelle (https://science.mnhn.fr/) and Chinese Virtual Herbarium (http://www.cvh.org.cn/).

### DNA extraction and sequencing

Total DNA was extracted from 17 silica-gel dried leaf pieces collected in the field. DNA extraction was performed using the CTAB method (Doyle and Doyle 1987) with minor modifications described in Toyama et al. (2015). Two chloroplast DNA barcode regions, *rbcL* and *matK*, were amplified and sequenced following published protocols (Kress et al. 2009, Dunning and Savolainen 2010). In addition, the internal transcribed spacer (ITS) region was sequenced using the protocol of Rohwer et al. (2009) with a minor modification in PCR amplification using the Tks GflexTM DNA Polymerase (Takara Bio Inc., Japan).

### Phylogenetic analysis

A total of 16 accessions representing 15 species of *Lithocarpus*, collected throughout Vietnam, were analysed (Table 1). In addition, *Trigonobalanus verticillata* Forman was used as an outgroup in the phylogenetic analysis. The sequence alignment was performed by ClustalW with default parameters implemented in MEGA v 7.0.25 (Kumar et al. 2016) and subsequently adjusted manually.

**Table 1.** List of taxa used in this study with vouchers and GenBank accession number.

Species	Vouchers	GenBank accession number		
		*rbcL*	*matK*	ITS
*Lithocarpus aggregatus*	*Tagane et al. V6288* (DLU, FU)	LC318967	LC318550	MF770309
*Lithocarpus bidoupensis*	*Tagane et al. V4320* (DLU, FU, VNM)	LC318961	LC318547	KY940070
*Lithocarpus coalitus*	*Tagane et al. V4191* (DLU, FU, VNM)	LC318959	LC318545	MF770305
*Lithocarpus dahuoaiensis*	*Ngoc et al. V3194* (DLU, FU, HN, K, KYO, P, VNM)	LC318953	LC318551	KY436002
	*Ngoc et al. V5404* (DLU, FU)	LC318964	LC318548	MF770307
*Lithocarpus gigantophyllus*	*Ngoc et al. V3185* (DLU, FU)	LC318951	LC318538	MF770299
*Lithocarpus hancei*	*Ngoc et al. V5111* (DLU, FU)	LC318963	LC318970	MF952868
*Lithocarpus hongiaoensis*	*Ngoc et al. V3235* (DLU, FU)	LC318956	LC318542	KY851759
*Lithocarpus lemeeanus*	*Tagane et al. V4273* (DLU, FU)	LC318960	LC318546	MF770306
*Lithocarpus licentii*	*Ngoc et al. V3205* (DLU, FU)	LC318954	LC318540	MF770301
*Lithocarpus longipedicellatus*	*Nguyen et al. V3813* (DLU, FU)	LC318958	LC318544	MF770304
*Lithocarpus ombrophilus*	*Yahara et al. V3000* (DLU, FU)	LC318949	LC318420	MF770297
*Lithocarpus pseudomagneinii*	*Ngoc et al. V3223* (DLU, FU)	LC318955	LC318541	MF770302
*Lithocarpus stenopus*	*Ngoc et al. V3187* (DLU, FU)	LC318952	LC318539	MF770300
*Lithocarpus vinhensis*	*Nguyen et al. V3787* (DLU, FU)	LC318957	LC318543	MF770303
*Lithocarpus vuquangensis*	*Yahara et al. V5743* (DLU, FU)	LC319671	LC319670	KY786083
*Trigonobalanus verticillata*	*Yahara et al. V5764* (DLU, FU)	LC318965	LC318549	MF770308

Bayesian Inference (BI) of phylogeny was performed on the concatenated data set of three genes (*rbcL*, *matK* and ITS) using MrBayes v. 3.2 (Huelsenbeck and Ronquist 2001, Ronquist et al. 2012). The hierarchical likelihood ratio test (hLRT) and Akaike Information Criterion (AIC) were used to select the best model of evolution using MrModeltest v. 2.3 (Nylander 2004). The nucleotide substitution model was set to GTR+γ as selected by MrModeltest. Four independent Markov Chain Monte Carlo (MCMC) runs of four chains each were run for 10,000,000 generations sampling every 1,000 generations. The programme Tracer v. 1.6 (Rambaut et al. 2014) was used to examine marginal prior and posterior densities of MCMC outputs. Each run produced 10,001 trees and a relative burnin of 25% was used for diagnostics. Consequently, 7,501 trees of each run were sampled to generate the summary tree and posterior probabilities distributions. The summary tree was visualised and edited with FigTree v1.4.3 (http://tree.bio.ed.ac.uk/software/figtree/).

## Results

The morphological comparison showed that *Lithocarpus vuquangensis* is most similar to *L. nantoensis* (Hayata) Hayata distributed in Taiwan, in having entire leaf margin, mostly solitary, rarely 2 or 3 clustered cupules, cupules not completely enclosing nut and glabrous nut. The Vietnamese species sharing the above diagnostic feature of *L. vuquangensis* are *L. hongiaoensis, in ined.* (Ngoc et al. in review) and *L. vinhensis* A. Camus. However, the new species is clearly different from all three in the following points: *L. vuquangensis* is distinguished from *L. nantoensis* by its fewer secondary veins (7–10 pairs vs. 10–15 pairs), shorter infructescences (4–7 cm long vs. 16 cm long), longer fruiting stalks (4–6 mm long vs. almost sessile), larger nut size (1.7–2.0 cm high by 2.1–2.4 cm in diam. vs. 1.4–1.7 cm high by 1.5–1.6 cm in diam.) and larger basal scar of the nut (ca. 1.1 cm in diam. vs. 0.5–0.8 cm in diam.). *Lithocarpus vuquangensis* is distinct from *L. hongiaoensis* by its shorter petioles (1–1.5 cm long vs. 2.1–3 cm long), shorter infructescences (4–7 cm long vs. 10 cm long), longer fruiting stalks (4–6 mm long vs. almost sessile), arrangement of scales on the cupule (scales arranged into concentric rings vs. imbricate, not forming rings) and larger nut size (1.7–2.0 cm long, 2.1–2.4 cm in diam. vs. 0.6–0.8 cm long, 1.2–1.5 cm in diam.). The new species differs from *L. vinhensis* in having fewer secondary veins (7–10 pairs vs. 11–12 pairs), shorter infructescences (4–7 cm long vs. 10 cm long) and larger nut size (1.7–2.0 cm long, 2.1–2.4 cm in diam. vs. 0.9–1 cm long, 1 cm in diam.). A more detailed comparison amongst these four species is shown in Table 2.

**Table 2.** Morphological comparison of *Lithocarpus vuquangensis* with three related species: The measurements of *L. nantoensis* is derived from Hayata (1911), Liao (1996), Huang et al. (1999) and from digitised type specimen image (*Kawakami & Mori 1157*, TI); The measurements of *L. vinhensis* and *L. hongiaoensis* are derived from Camus (1948) and Ngoc et al. (in review), respectively.

Characters	*L. vuquangensis*	*L. nantoensis*	*L. hongiaoensis*	*L. vinhensis*
Leaf margin	Entire	Entire	Entire	Entire
Leaf surface	Glabrous adaxially, adaxially white farinose	Abaxially glaucous to light green and with adherent, waxy scalelike trichomes	Glabrous upper, adherent waxy scale abaxially	Glabrous adaxially, covered with very short white villi abaxially
Leaf blade size	7.5–11 × 2.3–3.6 cm	12–16 × 2.5–3.5 cm	9.6–14.5 × 2.5–3.8 cm	7.5 cm × 3 cm
Petiole length	1–1.5 cm long	0.7–1.3 cm long	2.1–3 cm long	1 cm long
Number of secondary veins	7–10 pairs	10–15 pairs	8–11 pairs	11–12 pairs
Infructescences length	4–7 cm long	16 cm long	12.5–16.5 cm long	10 cm long
Fruiting stalk length	4–6 mm long, 4–7 mm in diam.	Almost sessile	Sessile to 2 mm long	5–6 mm long
Cupule	Solitary, 0.6–0.9 cm high by 1.8–2.2 cm in diam.	Solitary, 1.2–1.5 cm in diam.	Solitary, 1–1.2 cm high by 1.8–2.1 cm in diam.	Solitary, 1.2–1.3 cm high by 0.8–1 cm in diam.
Scale arrangement	Arranged into concentric rings	Arranged into concentric rings	Imbricate	Arranged into concentric rings
Nut size	1.7–2.0 cm high by 2.1–2.4 cm in diam.	1.4–1.7 cm high by 1.5–1.6 cm in diam.	0.6–0.8 cm high by 1.2–1.5 cm in diam.	0.9–1 cm high by 1 cm in diam.
Nut enclosure by cupule	Only basal to 1/4 of the nut	Only basal part of the nut	Enclosing ca. 1/3–1/2 of the nut	Enclosing ca. 1/3–1/2 of the nut
Basal scar of the nut	Concave, ca. 1.1 cm in diam.	Concave, 0.5–0.8 cm in diam.	Slightly concave, 1.2–1.4 cm in diam.	Nearly flat

**Figure 2.**
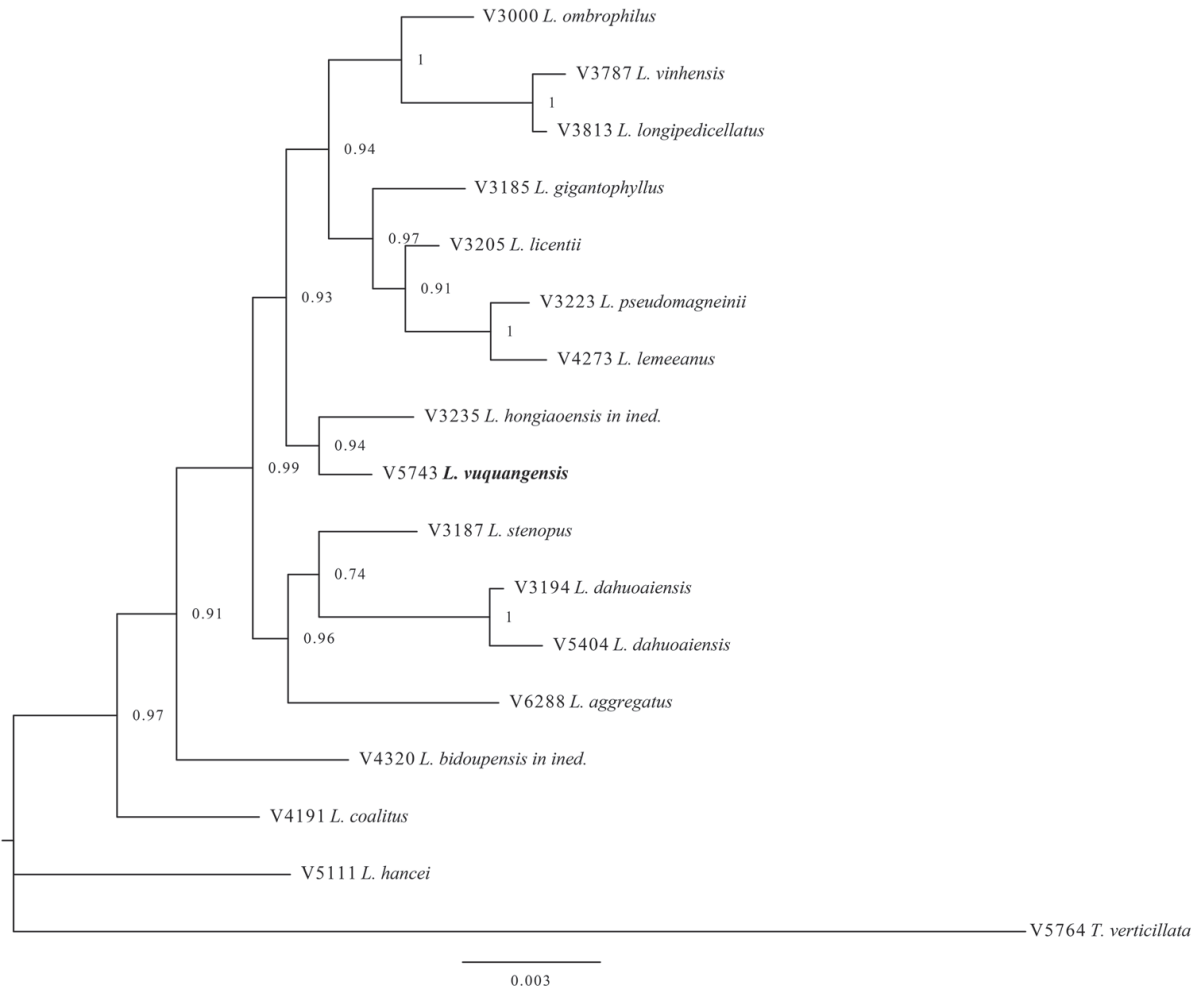
Bayesian phylogeny estimate of 15 taxa of *Lithocarpus* and one *Trigonobalanus verticillata* (as an outgroup) based on combined *rbcL*, *matK* and ITS sequences. Branches are labelled with posterior probabilities greater than 0.7.

In the molecular phylogenetic tree (Fig. 2), *L. vuquangensis* is sister to *L. hongiaoensis* with the posterior probability of 0.94. One nucleotide substitution in *rbcL*, six in *matK* and six in ITS were found between these two species. On the other hand, *L. vinhensis*, another Vietnamese species most similar to *L. vuquangensis*, is placed in a separated clade which includes *L. longipedicellatus, L. ombrophilus, L. gigantophyllus, L. licentii, L. pseudomagneinii* and *L. lemeeanus*, with a posterior probability 0.93.

Both *Lithocarpus vuquangensis* and *L. vinhensis* were collected in Vu Quang National Park, but these two species occur at different altitudes: *L. vuquangensis* was found between 1,500 m and 1,700 m altitude, while *L. vinhensis* was found at a lower elevation, below 1,100 m.

## Discussion

Phylogenetically, *L. vuquangensis* is sister to *L. hongiaoensis in ined.* collected from Lam Dong Province located in southern Vietnam. These two species are morphologically distinguished in their length of infructescences and fruiting stalk, the arrangement of cupule bracts, nut size and other characteristics as summarised in Table 2. Further molecular phylogenetic studies, using additional DNA markers, are needed to clarify the relationship between *L. vuquangensis* and *L. hongiaoensis*. However, morphological differences are sufficiently distinct to distinguish them as different species.

*Lithocarpus vuquangensis* is also morphologically similar to *L. vinhensis* in having an entire leaf margin, solitary cupules not completely enclosing nut, scales arranged into concentric rings and glabrous nut, but these two species are not closely located in the phylogeny. This morphological similarity may have evolved in the similar habitat of the montane evergreen forest in Vu Quang National Park. Whereas *L. vuquangensis* and *L. vinhensis* were collected at 1,518 m and 1,062 m, respectively, altitudinal distributions of the two species may overlap in the montane evergreen forest.

The morphological comparison provided evidence to distinguish *L. vuquangensis* from a Taiwanese species, *L. nantoensis*, although the relationship between them remains to be clarified by further molecular phylogenetic studies.

## Taxonomy

### Lithocarpus vuquangensis

Taxon classificationPlantaeFagalesFagaceae

Ngoc & Hung sp. nov.

urn:lsid:ipni.org:names:60475914-2

Figure 3

#### Diagnosis.

Similar to *Lithocarpus nantoensis*, *L. hongiaoensis* and *L. vinhensis*, but distinguished from *L. nantoensis* mainly by its fewer secondary veins, shorter infructescences, longer fruiting stalk, larger nut size and larger scar size of the nut, from *L. hongiaoensis* by its much shorter petioles and infructescences, longer fruiting stalk, scales united into concentric rings and much larger nut size and from *L. vinhensis* by having fewer secondary veins, shorter infructescences and much larger nut size (Table 2).

#### Type.

VIETNAM. Ha Tinh Province, Vu Quang National Park, in lower montane forest, along trail to the summit of Mt. Rào Cô, alt. 1518 m, 18°12'12.2"N, 105°23'15.3"E, 22 June 2016, *Yahara T., Nguyen Van Ngoc, Toyama H., Tagane S., Okabe N., Nguyen Viet Hung V5743* (holotype: KYO!; isotypes: DLU!, FU!, HN!, K!, P!, VNM!).

#### Description.

Trees, to 20 m tall; young branches mostly glabrous, yellowish *in vivo*, reddish-brown *in sicco*. Leaves alternate, spirally arranged, blade narrowly elliptic to lanceolate, 7.5–11 × 2.3–3.6 cm, crunchy, glabrous adaxially, white farinose abaxially, apex long acuminate, acumen up to 1.2 cm long, base cuneate to attenuate, margin entire and wavy; midrib flat or slightly prominent near base adaxially, prominent abaxially, greenish-yellow *in vivo*, reddish-brown *in sicco*, secondary veins 7–10 pairs, at an angle of 40–50 degrees from the midrib, prominent abaxially, tertiary veins scalariform, faintly visible to invisible on both sides; petiole 1–1.5 cm long, glabrous, terete. Male inflorescence a spike, 7–8.5 cm long. Male flower solitary; calyx 6-lobed, lobes ovate, 0.5–0.6 mm × 0.4–0.5 mm, pubescent on both surfaces; stamens 12, 0.7–0.9 mm long, anthers 0.1–0.15 mm long. Infructescences erect, woody spike, up to 7 cm long, axis ca. 2 mm thick at base, greyish-brown, lenticellate. Cupule solitary, broadly obconical to saucer-shaped, 1.4 cm long, 1.8 cm in diam., enclosing only basal to 1/4 of the nuts; scales triangular, arranged into 4–5 concentric rings, apex shortly acuminate, densely covered with tawny minute hairs; fruiting stalk ca. 4–6 mm long, 4–7 mm in diam. Nut obovoid or globose, 1.7–2.0 cm long, 2.1–2.4 cm in diam., glabrous, dehiscent; basal scar concave, ca. 1.1 cm in diam.

**Figure 3.**
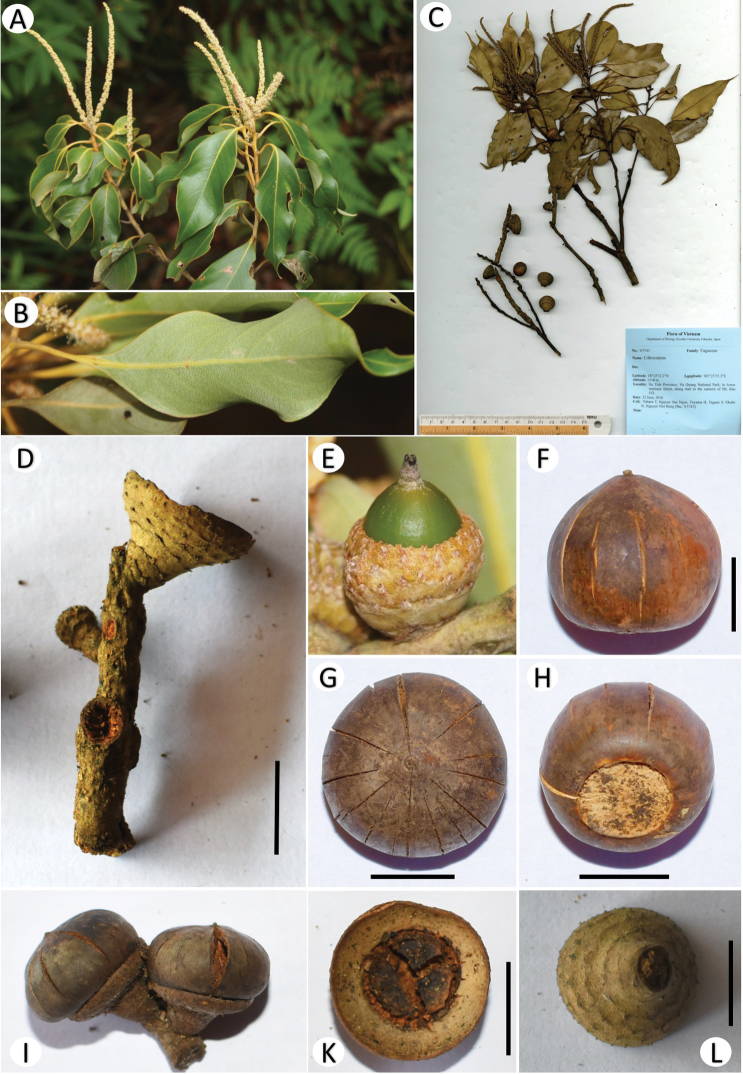
*Lithocarpus vuquangensis* Ngoc & Hung: **A** Branch with male inflorescences **B** Lower leaf surface **C** Holotype (KYO) **D** Spike with cupule **E** Young acorn **F–H** Side view, top view and scar of the mature nut, respectively **I** A part of infructescence **K, L** Inside and outside of the cupule. **C, D, F–L** From *Yahara et al. V5743*. Scale bars: 2 cm (**D**), 1 cm (**F–H, K, L**).

#### Phenology.

Mature fruits were collected in June.

#### Distribution.

Vietnam (so far known only from Vu Quang National Park, Ha Tinh Province) (Figure 1).

#### Etymology.

The specific epithet is derived from its type locality, Vu Quang National Park.

#### GenBank accession No.

*Yahara et al. V5743*: LC319671 (*rbcL*), LC319670 (*matK*), KY786083 (ITS).

#### Preliminary conservation status.

Critically Endangered (CR). In the field observation, less than 10 individuals were found along the trail to the summit of Mt. Rào Cô, in lower montane forest. The habitat is inside the protected areas of Vu Quang National Park, but based on criterion D of the IUCN Red List criteria (IUCN 2012), this species is qualified as CR. Further intensive inventories are needed to find additional populations in Vu Quang National Park and its surrounding areas.

## Supplementary Material

XML Treatment for Lithocarpus vuquangensis
